# Construction and utilization of Fe_3_O_4_@Al^3 +^ immobilized laccase for enhancing organic diethylstilbestrol removal: A multi-spectroscopy and molecular docking investigation

**DOI:** 10.1016/j.csbj.2025.07.044

**Published:** 2025-08-13

**Authors:** Tianzhu Guan, Chenxi Ren, Yining Feng, Canfeng Bian, Huaxiang Li, Qingling Wang

**Affiliations:** a School of Food Science and Engineering, Yangzhou University, Yangzhou, 225127, China; b College of Chemistry and Environmental Science, Laboratory of Xinjiang Native Medicinal and Edible Plant Resources Chemistry, Kashi University, Kashi, China; c Key Laboratory of Catering Food Safety and Systematic Monitoring for Jiangsu Province Market Regulation

**Keywords:** Laccase, Fe₃O₄@Al³ ⁺, Degradation, Molecular docking, Immobilization, Diethylstilbestrol

## Abstract

As one of the endocrine-disrupting compounds (EDCs), diethylstilbestrol (DES) poses significant risks to ecosystems and human health. This study reports the development of a magnetic nanocomposite (Fe₃O₄@Al³⁺@laccase) *via* glutaraldehyde (GA) crosslinking to enhance DES degradation. The single factor experiments revealed that the optimum immobilization conditions were: temperature 40℃, pH 5, enzyme concentration 1.0 mg/mL, and immobilization time 1.0 h. Under these conditions, Fe₃O₄@Al³ ⁺@laccase achieved 91.71 %±2.95 % DES degradation within 12 h, as validated by HPLC, retaining 31.29 %±1.99 % of its initial catalytic activity after 10 reuse cycles. Notably, the immobilized laccase exhibited superior stability in organic solvents compared to free laccase. Fourier-transform infrared (FTIR) analysis confirmed that laccase was successfully conjugated onto Fe₃O₄@Al³ ⁺. Scanning electron microscopy and transmission electron microscopy revealed that the immobilization process preserved the original morphology and crystalline structure of Fe₃O₄@Al³ ⁺. Multi-spectroscopic analyses, including enzyme kinetics, fluorescence, and three-dimensional (3D) spectroscopy, elucidated the binding affinity and conformational changes between laccase and DES. Molecular docking simulations predicted a binding free energy of −6.4 kJ·mol⁻¹ , indicating that the conformation stability between laccase and surrounding free amino acids was mainly maintained *via* van der Waals, pi-pi T-shaped, Pi-Donor Hydrogen Bond, and Pi-Alkyl. This work provides proof-of-concept for using Fe₃O₄@Al³ ⁺ as a reusable magnetic support for laccase, offering a promising strategy for DES degradation and guiding the design of advanced nanomaterials for environmental bioremediation.

## Graphical Abstract

**Figure:**
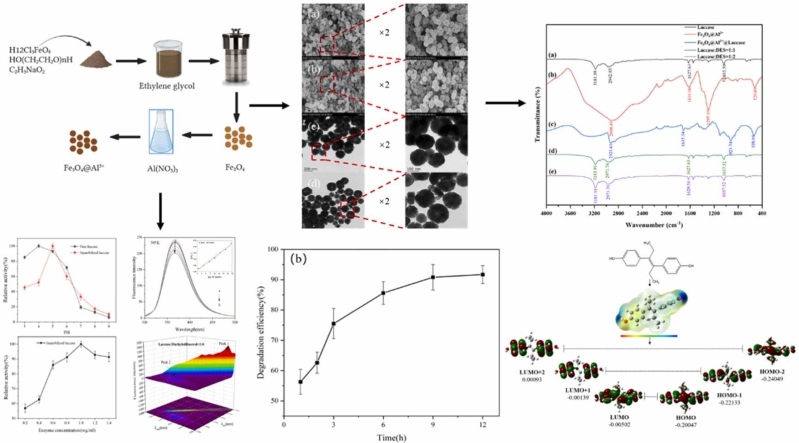
As a food born endocrine disruptors, diethylstilbestrol (DES) has potential harmful effects to the natural environment and human health. In order to safely and efficiently remove of DES, Fe3O4@Al^3+^@laccase were prepared under the optimum conditions: temperature 40℃, pH 5, enzyme concentration 1.0 mg/mL and immobilization time 1.0 h. The degradation efficiency of DES by Fe3O4@Al^3+^@laccase reaches 91.71 % after 12 h and the activity of Fe3O4@Al^3+^@laccase can still reach 31.29 % after being reused for 10 times. All in all, this study provided a useful tool for the exploration of new magnetic materials for the immobilized laccase and DES degradation.

## 1 Introduction

Diethylstilbestrol [(*E*)-4,4-(1,2-diethyl-1,2-ethenediyl) bisphenol] (DES), a synthetic nonsteroidal estrogen, is widely utilized in clinical and agricultural applications, including miscarriage prevention, prostate cancer treatment, and growth promotion in poultry and fish [1]. Unlike natural estrogens, DES exhibits greater chemical stability and a longer biological half-life [2], [3], [4]. Excessive use of DES can lead to its accumulation in humans and livestock, leading to adverse health effects. DES residues have been detected in fish, cattle, sheep, and other animals across multiple countries [5], [6]. Beyond direct exposure, DES can biomagnify through the food chain, ultimately accumulating in humans and posing risks to public health. Extensive toxicological and carcinogenicity research has demonstrated that prolonged exposure to DES significantly elevates the risk of malignant tumors in exposed fetuses and young females during developmental stages. Given these findings, the development of safe and efficient strategies for DES removal is critically important [7]. This study highlights the urgent need for innovative approaches to mitigate environmental and health risks associated with DES contamination, particularly in food systems.

Compared with conventional chemical, physical, and biological methods, enzymatic treatment has garnered significant attention for its selectivity, high efficiency, mild reaction conditions, and environmental sustainability [8], [9]. Among these enzymes, laccase—a multicopper oxidase produced by certain bacteria, fungi, and plants—has gained prominence for its eco-friendly nature, low energy requirements, and substrate specificity [10]. Structurally, laccase is a polyphenol oxidase containing four copper ions, classified within the ceruloplasmin oxidase family and typically present as a monomeric glycoprotein. It catalyzes the oxidation of a wide range of organic substrates, using molecular oxygen as an electron acceptor and produces water as its only byproduct. Recent studies have demonstrated laccase’s broad-spectrum catalytic capabilities in degrading phenolic compounds, polyphenols, chlorophenols, polyamines, dyes, aromatic substrates, and select inorganic compounds [11], [12]. However, its practical application is hindered by its susceptibility to denaturation under extreme pH, temperature, and organic solvent conditions [13]. To address these limitations, immobilization of laccase onto suitable carriers has been explored to enhance its operational stability and reusability, thereby expanding its utility in industrial and environmental remediation processes [14], [15].

Immobilization involves the covalent or physical coupling of catalysts to an insoluble carrier matrix to maintain structural integrity and enhance functional stability [16]. Methods for laccase immobilization include adsorption, covalent bonding, cross-linking/self-immobilization, and encapsulation. Compared to free enzymes, immobilized enzymes offer significant economic advantages, primarily attributed to their enhanced operational stability, reusability, and ease of separation from reaction mixtures. These attributes have propelled extensive research and industrial adoption of immobilized enzymes across diverse sectors, including medicine, the food industry, chemical manufacturing, and environmental remediation [17]. Magnetite (Fe₃O₄), a widely utilized magnetic material, is distinguished by its high saturation magnetization, superparamagnetic properties, and excellent biocompatibility [18], [19], [20]. Recent advancements have focused on modifying Fe₃O₄ nanoparticles through cation doping to improve their physicochemical properties. Aluminum ions (Al³⁺), given their physiological, biological, and electrochemical significance, have emerged as a critical dopant. The synthesis of the Fe₃O₄@Al³ ⁺ nanocomposites represents a promising strategy to integrate the advantageous properties of both components. This hybrid material not only provides a stable and biocompatible matrix for laccase immobilization but also creates an optimal microenvironment that preserves the enzyme’s catalytic efficiency. The Fe₃O₄@Al³ ⁺ carrier facilitates precise enzyme orientation and substrate accessibility, thereby maximizing catalytic performance while ensuring robustness against harsh reaction conditions [21], [22].

In summary, this study successfully synthesized Fe₃O₄@Al³ ⁺@laccase to optimize immobilization conditions and evaluate its catalytic performance toward DES. FTIR, SEM, TEM, and multi-spectroscopic analyses were employed to characterize the immobilized enzyme system and elucidate the binding mechanism between laccase and DES. Single-factor experiments identified critical parameters influencing enzymatic activity, while HPLC and molecular docking revealed DES degradation efficiency and interaction dynamics. This work advances understanding of enzymatic degradation of phenolic pollutants and demonstrates its potential for wastewater treatment and pesticide removal, contributing to sustainable pollution control.

## 2 Materials and methods

### 2.1 Chemical materials

Sodium alginate, glutaraldehyde (GA), laccase and ABTS were acquired from Yuanye Biotechnology (Shanghai). DES, FeCl_3_•6 H_2_O, HO(CH_2_CH_2_OnH), C_2_H_6_O_2_, and CH_3_COONa were acquired from Aladdin Biochemical Technology (Shanghai). S-4800 Ⅱ field emission scanning electron microscope (Hitachi, Japan) and Tecnai 12 transmission electron microscope (Netherlands) were used to record the SEM and TEM characteristics. J-810 circular dichroism spectrometer (JASCO, Japan) and fluorescence spectrophotometer F320 (Shanghai) were used to record fluorescence spectrum and 3D spectra. All experiments were carried out in triplicate.

### 2.2 Preparation of Fe_3_O_4_@Al^3+^

The synthesis of Fe₃O₄@Al³ ⁺ nanoparticles was adapted from the method described by Liu et al. [23], [24]. In brief, 2.7 g of FeCl₃·6H₂O was completely dissolved in 80 mL ethylene glycol. Subsequently, 2.0 g of polyethylene glycol (PEG) and 7.2 g of CH₃COONa were incorporated into the solution. The mixture was vigorously stirred for 30 min and then transferred to a PTFE-lined autoclave. The reaction was maintained at 200°C for 8 h. After cooling to room temperature, the products were dispersed in 80 mL of 1 mol/L Al(NO₃)₃ solution and stirred at 35°C and 200 rpm for 12 h. The Fe₃O₄@Al³ ⁺ nanoparticles were magnetically separated and extensively washed with deionized water to remove unreacted precursors. Finally, the nanoparticles were collected, rinsed sequentially with ethanol (EtOH) and distilled water, and dried under vacuum at 50°C for further use.

### 2.3 Preparation of Fe_3_O_4_@Al^3+^@laccase

Fe₃O₄@Al³ ⁺ magnetic nanoparticles were immobilized with laccase using a cross-linking approach adapted from previously reported methods [25]. Briefly, Fe₃O₄@Al³ ⁺ magnetic nanoparticles were incubated in a laccase solution prepared in Tris-HCl buffer (50 mM, pH 5.0). Immobilization was performed in the presence of 2.5 % GA to facilitate covalent attachment of the enzyme to the nanoparticle surface. Following completion of the immobilization process, the Fe₃O₄@Al³ ⁺@laccase conjugate was magnetically separated from the reaction mixture and washed three times to remove unbound laccase and residual GA. The immobilized enzyme complex was collected, stored at 4°C, and used for subsequent experiments [26].

### 2.4 Single factor experiments

The effects of various factors on the activities of free laccase and Fe₃O₄@Al³ ⁺@laccase were investigated across four parameters: temperature (30.0℃-90.0℃), pH, enzyme concentration, and immobilization time. pH-dependent activity was assessed by adjusting the laccase solution pH from 3.0 to 9.0 [27]. The influence of enzyme concentration on immobilized laccase activity was determined by varying the initial laccase concentration (1–7 mg/mL) during the immobilization process [28]. Additionally, the impact of immobilization time was examined under constant conditions to optimize the procedure.

### 2.5 Laccase enzyme activity

Laccase activity was assayed using ABTS as the substrate. The reaction system contained 1 mL of free laccase or 100 mg of Fe₃O₄@Al³ ⁺@laccase, 1 mL of ABTS solution, and 1 mL of buffer (50 mM, pH 5.0). Controls included ABTS and buffer solutions without laccase, with water replacing the enzyme. Absorbance at 420 nm was monitored and the laccase activity was calculated based on the following formula [29]:

(1) $$\text{Free laccase activity}\text{（}\text{U}/\text{mL}\text{）}=\frac{∆\text{A}\times {\text{V}}_{1}\times 10^{3}}{\text{t}\times {\text{V}}_{2}\times \text{ε}}\;$$

Where: ∆A——the change of absorbance within the reaction time, t; T—reaction time, min; V_1_—total volume of solution, mL; V_2_—the volume of added laccase solution, mL; ε—molar extinction coefficient of ABTS^+^, 360 mmol^−1^ cm^−1^.

(2) $$\text{Immobilized laccase activity}\text{（}\text{U}/\text{mL}\text{）}=\frac{∆\text{A}\times {\text{V}}_{\mathrm{Total}}\times 10^{3}}{\text{t}\times \text{m}\times \text{ε}}\;$$

Where: ∆A—the change of absorbance within the reaction time, t; T— reaction time, min; V_Total_— total volume of solution, mL; m— mass of Fe_3_O_4_@Al^3+^@laccase added, g; ε— molar extinction coefficient of ABTS^+^, 360 mmol^−1^ cm^−1^.

### 2.6 SEM and TEM

For sample preparation, all solid samples were finely ground prior to analysis. A small amount of powder was mounted on double-sided tape attached to the sample stage, with excess powder carefully removed by blowing. The surface morphology and particle size of Fe₃O₄@Al³ ⁺ and Fe₃O₄@Al³ ⁺@laccase were analyzed using SEM. For TEM, synthetic materials were dispersed in ultrapure water to form a homogeneous suspension following the method described by Gao et al. [30]. For Fourier-transform infrared (FTIR) spectroscopy, laccase and DES were mixed with varying ratios. The mixture was freeze-dried for 72 h, after which attenuated total reflectance (ATR) FTIR spectra were recorded [31]. Spectra were obtained for free laccase, immobilized Fe₃O₄@Al³ ⁺, Fe₃O₄@Al³ ⁺@laccase, and laccase-DES mixtures over a wavenumber range of 4000–400 cm⁻¹.

### 2.7 Enzyme kinetics

Laccase is known for its broad catalytic ability to degrade various phenolic compounds. DES, as a synthetic estrogen, contains a phenolic group similar to other phenolic compounds. Although ABTS and DES differ in their chemical structures, the shared phenolic group makes ABTS a representative model for studying the degradation of phenolic compounds, including DES. Therefore, initial reaction rate of laccase was determined by changing the concentration of ABTS from 0.05 to 1.00 mM (0.05, 0.10, 0.25, 0.50, 0.75, 1.00 mM). Thus, the kinetic parameters of laccase were determined. K_m_ and V_max_ were calculated as follows:

(3) $$\frac{1}{\text{ν}}=\frac{{\text{K}}_{\text{m}}}{{\text{ν}}_{\max}}\times \frac{1}{\left[\text{S}\right]}+\frac{1}{{\text{ν}}_{\max}}$$

where V was the initial reaction rates (mM/min), V_max_ represented the maximum reaction rate (mM/min), K_m_ represented Michaelis constant, and [S] represented the substrate concentration (mM).

### 2.8 Organic solvent stability

Stability of free laccase and immobilized laccase was evaluated by incubating both forms of the enzyme in various organic solvents, including EtOH, acetonitrile (ACN), and dimethyl sulfoxide (DMSO). Enzyme activity in water was set as 100 %, and the relative activity in each solvent was measured to assess stability [32].

### 2.9 Fluorescence quenching

The laccase was mixed with DES in certain proportions. The concentration of laccase in the system was set to 1.0 × 10^−6^ mol/L. The concentration gradient of DES was set to 0.5, 1, 1.5, 2, 2.5, 3 and 3.5 × 10^−6^ mol/L. The measurement temperature was set to 305, 310 and 315 K, the excitation wavelength was 280 nm, and the emission wavelength was 300–500 nm. Fluorescence determination formulas ((4), (5), (6), (7)) are as follows:

(4) F_0_/F= 1+K_sv_[Q]= 1+K_q_τ_0_[Q]

where K_sv_ represented the quenching constant, [Q] represented the concentration of quencher, K_q_ and τ_0_ respectively represented the quenching rate constant of biomolecules and the average life of protein at about 10^8^ s without quencher.

(5) log[(F_0_−F)/F]=logK_a_+nlog[Q]

where K_a_ and n represented the binding constant of laccase/DES and the number of binding sites per albumin molecule, respectively. We calculated the enthalpy change (δH) and entropy change (δS) to determine the interaction between laccase and DES, as shown below [33]:

(6) ln(K_2_/K_1_)= ΔH(1/T_1_−1/T_2_)

(7) lnK= −ΔH/RT+ ΔS/R

Where T represented the reaction temperature (K); K was the binding constant (L⋅mol^−1^); R = 8.314 J⋅K^−1^⋅mol^−1^.

### 2.10 3D fluorescence spectrum

The solutions with different concentration ratios of laccase and DES were added to a series of colorimetric tubes. The 3D spectrum of the emission wavelength in the range of 250–500 nm was measured at excitation wavelength of 280 nm.

### 2.11 Quantification of DES

A standard solution of DES was prepared with concentration gradients of 5, 10, 15, 20, and 25 mg/L, and a calibration curve was constructed by plotting concentration against peak area using high-performance liquid chromatography (HPLC). For the degradation experiment, 100 mg of immobilized laccase was added to a DES solution, and the reaction was conducted in a thermostatic shaker at 30°C and 120 rpm. Samples were collected at 1, 2, 3, 6, 9, and 12 h, followed by triplicate extractions with ethyl acetate. The organic phase was collected, dried, and evaporated using rotary evaporation. The residue was reconstituted in chromatographic methanol to a constant volume. The sample was filtered through a 0.22 μm microporous filter and analyzed using HPLC equipped with a fluorescence detector (Shimadzu LC-10 AT, Shimadzu, Tokyo, Japan) and a Zorbax SB C18 reversed-phase column (5 μm, 4.6 × 100 mm). The HPLC conditions were as follows: detection wavelength, 240 nm; flow rate, 1.0 mL/min; injection volume, 10 μL; column temperature, 30°C; and mobile phase, ACN:water (70:30, v/v). Degradation efficiency was calculated based on peak area comparisons.

### 2.12 Repeatability experiments

Fe₃O₄@Al³ ⁺@laccase prepared under optimal conditions was reused for degradation experiments. A 50 mg aliquot of Fe₃O₄@Al³ ⁺@laccase was immersed in 10 mL of DES solution and subjected to degradation in a thermostatic oscillator at 30°C and 120 rpm. After 15 min, the Fe₃O₄@Al³ ⁺@laccase was magnetically separated, rinsed with deionized water to remove residual DES, and reused for subsequent degradation cycles. Enzyme activity was normalized to the initial activity (defined as 100 %), and the retention of activity after each reuse cycle was determined to assess the reusability and stability of the immobilized enzyme system.

### 2.13 Molecular docking

Intermolecular interaction forces between laccase and DES were quantificationally investigated using AutoDock Vina and AutoDock Tools [34]. The structure of laccase was retrieved from the PDB database. The structure of DES was generated using Gauss View 6, followed by energy minimization. Docking parameters included a grid box size of 80 × 80 × 80 Å and a grid resolution of 0.4 Å. The docking process evaluated up to 200 candidate conformations to calculate free binding energy based on the scoring function. Visualization of the docking results was performed using PyMOL and Discovery Studio 2016 [35].

## 3 Results and discussion

### 3.1 Optimization of the immobilization condition

**Fig. 1:**
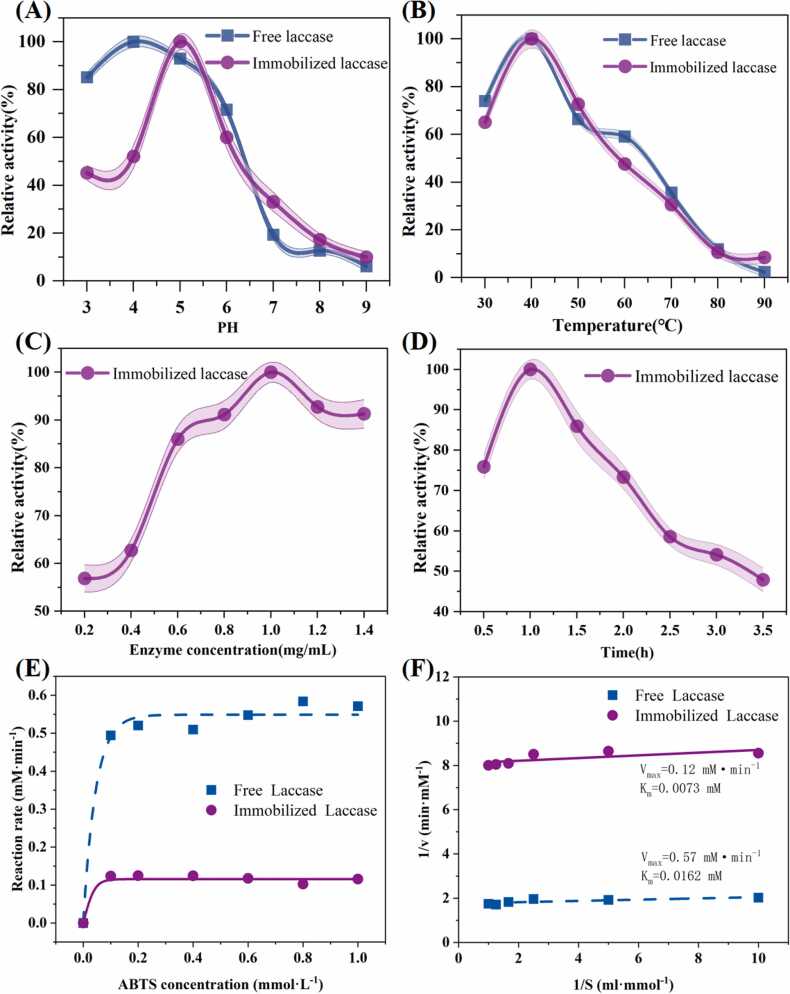
Effects of (A) pH, (B) temperature, (C) enzyme concentration, and (D) immobilized time on of free and Fe_3_O_4_@Al^3+^@laccase. (E-F) Enzyme kinetics of free and immobilized laccase: (E) Michaelis-Menten and (F) Lineweaver-Burk curves.

Operational stability of laccase under varying pH, temperature, concentration, and immobilization time is critical for its practical application in industrial processes. To evaluate the pH-dependent activity of free and immobilized laccase, experiments were conducted across a pH range of 3–9. Results revealed that free laccase exhibited maximum activity at pH 4.0, whereas Fe_3_O_4_@Al^3+^@laccase achieved its highest activity at pH 5.0 (Fig. 1A). Notably, Fe_3_O_4_@Al^3+^@laccase retained over 65 % of its relative activity within the pH range of 3–6, indicating a broader pH tolerance compared to free laccase. This pH insensitivity likely arises from the protective effect of the Fe_3_O_4_@Al^3+^ carrier, which stabilizes the enzyme structure and prevents denaturation under acidic or alkaline conditions [36]. The influence of temperature on enzyme activity was investigated using a range of 30–90°C. Both free and immobilized laccase displayed optimal activity at 40°C (Fig. 1B). The consistent optimal temperature for both forms suggests that the immobilization process does not alter the enzyme’s thermal stability. However, the immobilized laccase exhibited enhanced resistance to temperature fluctuations, likely due to the chelation between laccase and the Fe_3_O_4_@Al^3+^ carrier. This interaction may increase the activation energy required to maintain the enzyme’s optimal conformation for substrate binding, thereby preserving activity across a broader temperature range [37].

The effect of initial substrate concentration on enzyme activity was explored within a range of 0.5–2.0 mg/mL. Immobilization efficiency initially increased with concentration, peaking at 1.0 mg/mL, beyond which activity declined (Fig. 1C). This decline at higher concentrations is attributed to spatial hindrance caused by excessive laccase loading, which obstructs access to active sites on the Fe_3_O_4_@Al^3+^@laccase complex. These findings underscore the importance of optimizing substrate concentration to maximize enzyme performance. Finally, the impact of immobilization time on enzyme activity was assessed. Results demonstrated that 1 h was the optimal immobilization duration (Fig. 1D). Prolonged immobilization times likely induce structural changes in the enzyme or carrier, leading to reduced activity. This observation aligns with previous studies indicating that excessive immobilization periods may compromise enzyme integrity [38]. Future studies will focus on quantifying enzyme loading and specific activity to further validate the relationship between immobilization efficiency and activity decay [39], [40].

### 3.2 Enzyme kinetics

The enzymatic activity of laccase and its kinetic parameters were evaluated by adjusting the concentration of ABTS (Fig. 1D-F). The K_m_ values of free laccase and Fe_3_O_4_@Al^3+^@laccase were 0.0162 mM and 0.0073 mM, respectively. Correspondingly, the V_max_ values of free and Fe_3_O_4_@Al^3+^@laccase were 0.57 and 0.12 mM/min. The lower K_m_ value of Fe_3_O_4_@Al^3+^@laccase compared to free laccase indicates a higher affinity for the substrate. This enhanced affinity may be attributed to reduced steric hindrance and improved substrate diffusion following immobilization [41]. In contrast, the lower V_max_ value of the immobilized laccase suggests a reduction in catalytic efficiency. This observation is likely linked to the covalent stabilization of the enzyme at multiple binding sites, which may restrict conformational flexibility, or to structural modifications at the active site during the immobilization process. Notably, similar trends in K_m_ and V_max_ values have been reported in previous studies, such as those by Shokri et al. and Mateja et al. [39], reinforcing the validity of these findings. The combined analysis of K_m_ and V_max_ highlights a trade-off between substrate affinity and catalytic efficiency in immobilized enzymes. While the immobilization process enhances substrate binding (lower K_m_), it simultaneously introduces constraints that limit the enzyme’s ability to convert substrates into products (lower V_max_). This balance is critical for optimizing the practical application of immobilized laccase in bioremediation processes, particularly for the removal of organic pollutants such as DES.

### 3.3 Fluorescence quenching experiments

**Fig. 2:**
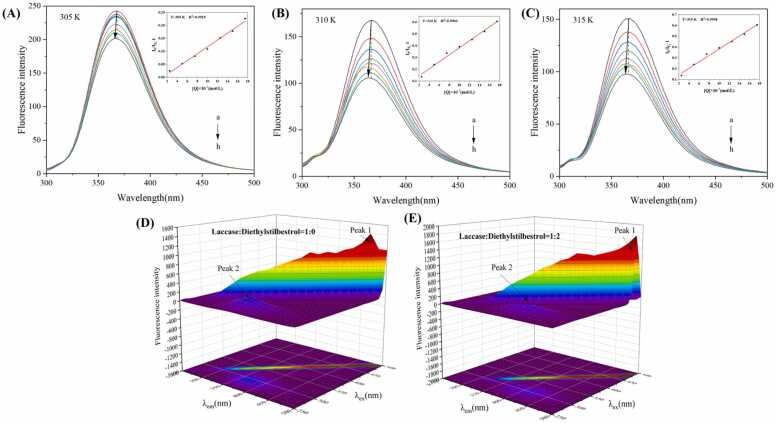
Fluorescence quenching spectra of laccase and DES system. (A) 305 K, (B) 310 K, (C) 315 K. (D, E) 3D fluorescent spectra of laccase in the absence and the presence of DES.

The investigation of quenching mechanism and binding mechanism between laccase and DES *via* fluorescence-based methods provides critical insights into ligand-receptor binding characteristics, including binding sites and binding constants. Additionally, the binding affinity between laccase and DES was quantified through fluorescence quenching experiments. The standard curve is shown in Supplementary Figure 1. From Fig. 2A-E, fluorescence intensity of laccase decreased with increasing DES concentration, indicating a quenching effect. The Stern-Volmer quenching constants (K_sv_) decreased with rising temperature, while the bimolecular quenching rate constant (K_q_) was approximately 10^13^ L·mol^−1^·s^−1^, which is 1000 times higher than the maximum diffusion collision rate constant of 2 × 10^10^ L·mol^−1^·s^−1^. This finding confirms that the quenching mechanism between laccase and DES is a typical static quenching process [42]. A red shift from 360 nm to approximately 371 nm was observed in the most intense emission peak of the fluorescence spectrum, indicating a conformational change in the tertiary structure of laccase. This shift suggests that the fluorophores within laccase transitioned from a hydrophobic environment to a more hydrophilic environment, likely due to the binding of DES to the laccase [43]. Table 1 reveals that the number of binding sites in the laccase-DES system was approximately 2, implying that DES interacts with laccase at two distinct binding sites. The strong binding affinity observed suggests that DES forms stable complexes with laccase, which is critical for its enzymatic degradation. Furthermore, thermodynamic parameters (ΔH^0^ and ΔS^0^) were calculated as −16.6856 kJ·mol^−1^ and 0.05932 kJ·mol^−1^·K^−1^, respectively (Table 1). The negative ΔH^0^ and positive ΔS^0^ indicate that the interaction between laccase and DES is primarily driven by electrostatic forces, with hydrogen bonding also playing a significant role in stabilizing the complex [44].

**Table 1:** laccase and DES system and linear correlation coefficient.

	**T/K**	**305**	**310**	**315**
Fluorescence quenching constant	K_sv_	1.3264 × 10^5^	2.9783*10^15^	2.8497*10^15^
	K_q_	1.3264*10^13^	2.9783*10^13^	2.8497*10^13^
	R^2^	0.9919	0.9903	0.9938
Binding constants and binding sites	Ka (L mol^−1^)	7.8505 × 10^5^	1.1748 × 10^5^	8.6516 × 10^5^
	n	1.1395	0.7459	0.9047
	Fitting equation (R^2^)	y = 1.1395 x + 5.8949 (0.9982)	y = 0.7459 x + 4.0700 (0.9967)	y = 0.9047 x + 4.9371 (0.9977)
Thermodynamic parameters	△H（KJ·mol^−1^）	−166.7256		
	△S（J·mol^−1^·K^−1^）	−0.5299		
	△G（KJ·mol^−1^）	−34.4193	−30.0879	−35.8023

### 3.4 3D fluorescence spectroscopy

As shown in Fig. 2D-E, distinct differences in peak positions from 3D fluorescence spectroscopy were observed between the two samples. Specifically, the fluorescence peaks of laccase were primarily located in the high-molecular-weight region (λ_em_ 350–400 nm, λ_ex_ 250–300 nm) and the low-molecular-weight region (λ_em_ 400–425 nm, λ_ex_ 275–325 nm). The characteristic fluorescence peaks of laccase are closely associated with the presence of Tyr and Trp residues, which are critical for maintaining the enzyme’s structural integrity and catalytic activity. Upon the addition of DES, a significant decrease in laccase fluorescence intensity occurred, indicating conformational changes in the enzyme when complexed with DES (Fig. 2E). This reduction in fluorescence intensity suggests that the binding of DES to laccase alters the microenvironment surrounding the Trp residues, increasing the polarity of their local environment [27], [45]. This observation is consistent with previous findings that ligand binding can induce structural rearrangements in enzymes, affecting their fluorescence properties. The results from the 3D fluorescence spectra are in good agreement with those obtained from fluorescence spectroscopy, collectively demonstrating that DES binding induces pronounced conformational changes and microenvironmental alterations in laccase. These findings provide critical insights into the molecular interaction mechanisms between laccase and DES, which are essential for understanding the enzymatic degradation process of this endocrine-disrupting compound.

### 3.5 FTIR analysis

**Fig. 3:**
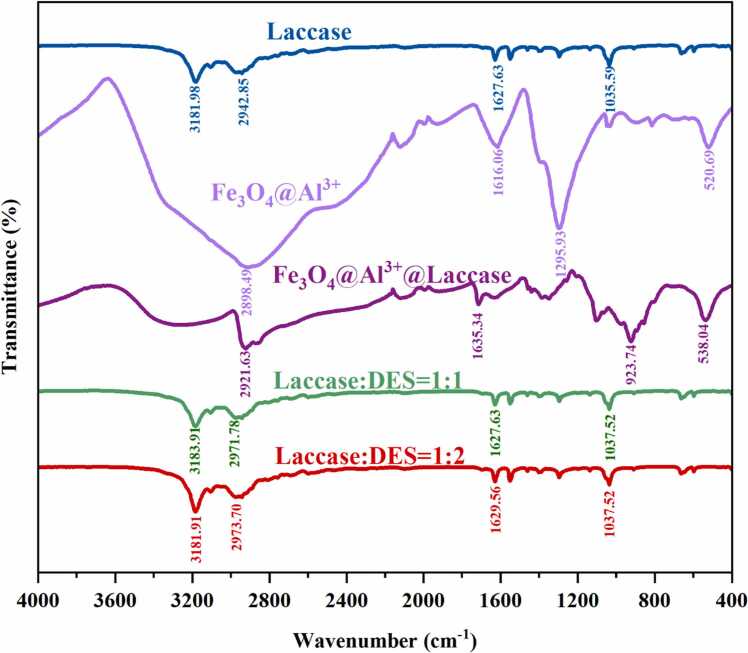
FTIR spectra of laccase and DES system.

The FTIR analysis was used to study chemical groups alterations of laccase, Fe_3_O_4_@Al^3+^, Fe_3_O_4_@Al^3+^@laccase and the mixture of DES and laccase with different proportions (Fig. 3). The peaks observed at 1627, 1295 and 1035 cm^−1^, representing the tensile vibration of carboxyl (C=O), C=C skeleton, C-OH and C-O-C in aromatic rings, respectively [46]. In addition, the broad peak at 3181 cm^−1^ could be attributed to the absorption of hydroxyl and water molecules [15]. Some characteristic absorption bands could be clearly observed to disappear or decay after DES was treated with cross-linked and embedded laccase. The strong vibration band observed at 520 cm^−1^ may be attributed to the tensile vibration of the prepared metal oxide in Fe_3_O_4_@Al^3+^, which confirmed the existence of the metal oxide [47]. A characteristic absorption band at 1161 cm⁻¹ corresponds to C–O–C skeletal vibrations of laccase. Compared to free laccase, the main peaks around 3181, 1627 and 1035 cm⁻¹ remained largely unchanged, indicating no loss or transfer of functional groups during immobilization and DES treatment. Compared with free laccase and Fe₃O₄@Al³ ⁺, the peaks of Fe₃O₄@Al³ ⁺@laccase at 3181.98 cm⁻¹ disappeared. This disappearance of the 3181.98 cm⁻¹ band, attributed to aromatic and aliphatic C–H stretching, further confirms successful immobilization of laccase on Fe₃O₄@Al³ ⁺ [48].

### 3.6 Structural characterization of Fe_3_O_4_@Al^3+^ and Fe_3_O_4_@Al^3+^@laccase

**Fig. 4:**
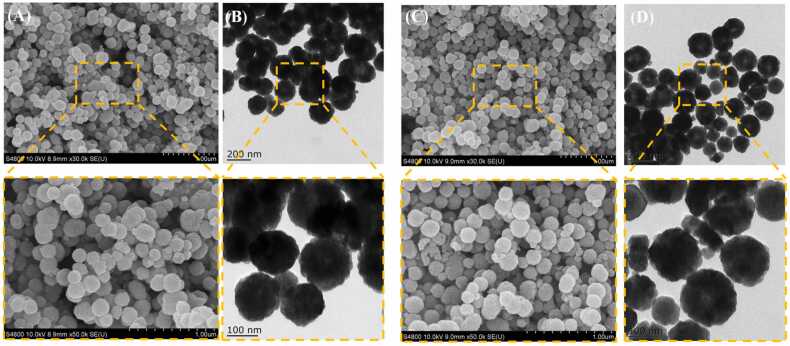
(A, B) SEM image of Fe_3_O_4_ and Fe_3_O_4_@Al^3+^@laccase. (C, D) TEM image of Fe_3_O_4_ and Fe_3_O_4_@Al^3+^@laccase.

The SEM and TEM of Fe_3_O_4_@Al^3+^ and Fe_3_O_4_@Al^3+^@laccase were shown in Fig. 4. The resulting nanoparticles were found to be spherical in shape, with sizes ranging from 100 to 200 nm. Fe_3_O4@Al^3+^ had rugged surface, extremely small size, large specific surface area and strong magnetism, which was beneficial to the separation of Fe_3_O4@Al^3+^laccase from substrate solution [27]. According to Fig. 4, the spatial network structure composed of Al^3+^ layer and Fe_3_O_4_ were relatively tight, without obvious boundaries. It could be observed from the surface of Fe_3_O_4_@Al^3+^ microspheres that the folding structure was compact and the richness was high which facilitates the contact with the substrate [49], [50]. Comparing the SEM and TEM images, the results showed that the immobilized laccase did not affect the structure and morphology of nanoparticles. As a result, Fe_3_O_4_@Al^3+^@laccase maintained the original excellent characteristics of the carrier [51].

### 3.7 Organic solvent stability

**Fig. 5:**
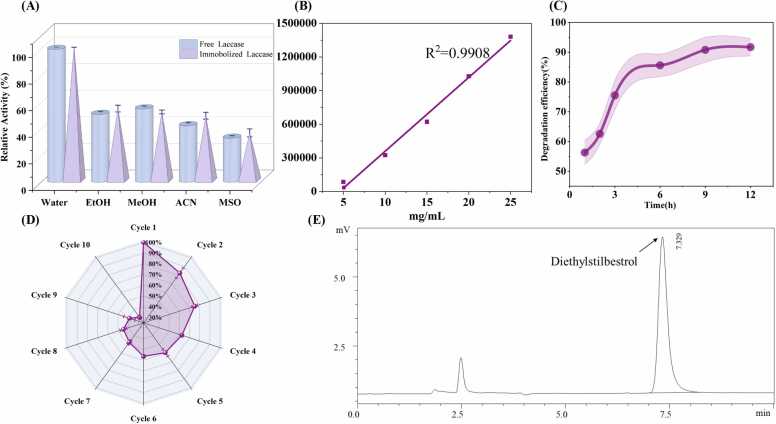
Stability and reusability of immobilized laccase and performance of its degradation. (A) Stability of free and immobilized laccase in different organic solvents. (B) Standard curve. (C) Degradation efficiency. (D) Reusability of immobilized laccase in several degradation process. (E) High-performance liquid chromatography of DES.

The enzyme activities in water were defined as 100 % because the water system exhibited the highest laccase activity among all tested solvents. The changes in enzyme activity within the organic solvent system were shown in Fig. 5A. Compared with pure water system, the laccase activity in organic solvent system was decreased. This phenomenon may be due to the organic solvents with strong polarity would seize the binding water of laccase molecules, thus affecting the hydration layer of the microenvironment of laccase molecules. Eventually, the catalytic activity of the laccase was reduced, and even the denaturation and inactivation of the laccase were caused [52].

### 3.8 Quantification of DES by HPLC method

Residual DES was quantified by HPLC analysis though degradation products were not identified. With the extension of time, the degradation efficiency of DES gradually increased from 56.3 %±4.12–91.7 %±2.95 % (Fig. 5C). This result showed that immobilized laccase has obvious degradation effect on DES. However, after 12 h, the removal efficiency of DES decreased gradually with the passage of time. This decline may be due to the reduction in DES concentration in the whole system. Additionally, the decreased efficiency could result from a loss of activity of the immobilized laccase after prolonged DES degradation [53]. Meantime, it was not excluded that the immobilized carrier limits the number of binding sites between laccase and DES, thus limiting the degradation effect of laccase [54].

### 3.9 Repeatability experiment

One of the main aspects that made laccase a useful candidate in industrial applications was its reusable potential. The stability of Fe_3_O_4_@Al^3+^@laccase was evaluated after repeated use for 10 times. The residual activity of immobilized laccase decreased with the increase of cycle times (Fig. 5D). To clearly reflect the activity decay trend during repeated use, the degradation efficiency of the first cycle was set as the 100 % baseline, and subsequent data represent relative ratios compared to the initial trial. The retention rate of residual activity was 31.29 %±1.99 % at the end of the tenth cycle, indicating a significant loss of activity over successive cycles. This phenomenon may result from several factors, including a decrease in enzyme activity due to prolonged use, denaturation, and shedding of the enzyme during the reaction, or losses during the magnetic separation process. To mitigate these issues, stronger immobilization techniques could be employed to reduce enzyme leaching. Optimizing magnetic separation conditions could also help reduce enzyme loss during recovery, ultimately improving the reusability of the enzyme in industrial applications [55].

### 3.10 Molecular electrostatic potential (MEP)

**Fig. 6:**
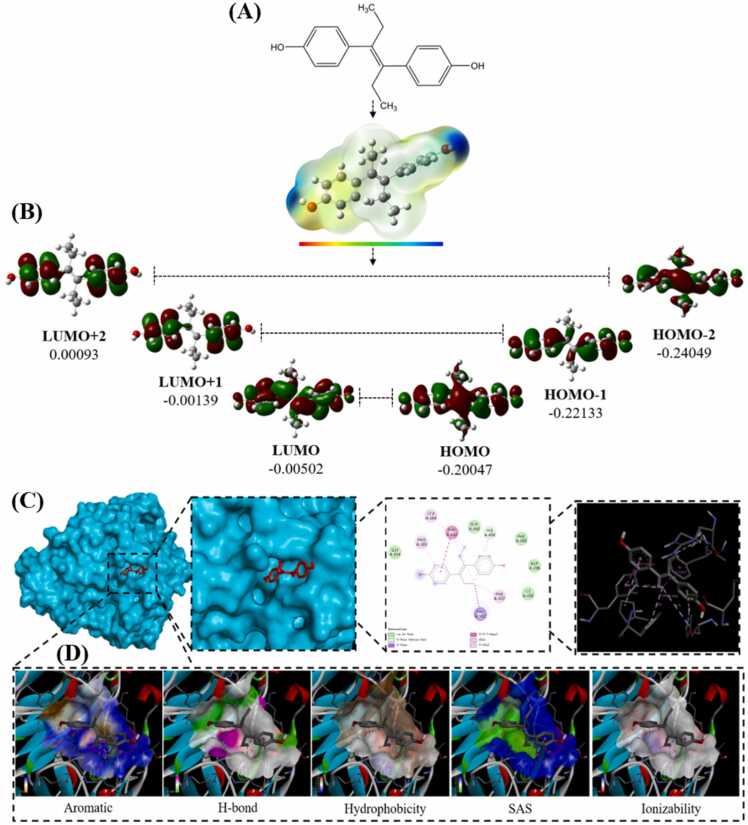
(A) Molecular electrostatic potential, (B) frontier molecular orbitals, and (C) docking conformations of laccase with DES.

Within the domain of molecular theoretical calculations, MEP serves as a powerful tool, capable of directly depicting the electron density, pinpointing chemical active sites, and illustrating the reactivity of atoms. This functionality is instrumental in identifying the electrophilic and nucleophilic regions of active compounds [56]. As depicted in Fig. 6 A, the MEP maps of five active compounds are constructed based on their optimized molecular geometries. In these visual representations, the negative (red) zones and positive (blue) zones correspond to regions of electrophilic and nucleophilic reactivity, respectively. Typically, the negative charge tends to congregate around the oxygen atom in a C=O group, while the positive charge is centered on the hydrogen atom within a methyl group. Consequently, the MEP of DES predominantly exhibits a positive character, with the methyl group’s hydrogen atom serving as the focal point of this positivity. More specifically, the MEP profile of DES provides a clear visualization of the interaction between primary receptors and associated ligands on the molecular surface. This detailed insight is invaluable for advancing our understanding of the interaction mechanisms between laccase and DES.

### 3.11 Frontier molecular orbital (FMO)

In the process of quantum chemical calculation, the size of HOMO and LUMO could be used to study the dynamic stability, energy performance and chemical reactivity of active molecules [57]. HOMO represents the electron-donating ability, and LUMO, as an electron acceptor, stranded for electron acquiring ability. The HOMO-LUMO energy calculation was carried out to determine the energy behavior of the compound. The 3D images of HOMO, HOMO+ 1, LUMO and LUMO+ 1 at the front edge of DES were shown in Fig. 6B. Red and green represented positive and negative phases respectively. The charge density is concentrated on the whole molecule for LUMO of DES. HOMO energy (E_HOMO_) and LUMO energy (E_LUMO_) are directly related to ionization potential and electron affinity, respectively. The energy gap (E_HOMO_–E_LUMO_) is the key index of molecular chemical stability for intramolecular charge transfer. Specifically, the E_HOMO_–E_LUMO_ of DES energy levels are 0.00093, −0.00139, −0.00502, −0.20047, −0.22133 and −0.24049 eV, respectively. Due to its low chemical reactivity, a large E_HOMO_–E_LUMO_ gap means polymer stability.

### 3.12 Molecular docking

To study the binding preference of binding sites on laccase to DES, DES was docked with laccase. Binding sites and parameters of each compound had the same definition [58]. The energy favorable conformation of DES binding posture was shown in detail in Fig. 6 C. The amino acid residues surrounding the ligand for DES were GLY^334^, PRO^391^, LEU^164^, PHE^332^, GLY^392^, HIS^458^, PHE^265^, ASP^206^, ILE^445^, PHE^337^, PHE^162^. Results of molecular docking between DES and laccase showed that carbonyl and aromatic ring had excellent binding score (−6.4 KJ⋅mol^−1^). The binding patterns of laccase and surrounding residues were studied. Fig. 6 showed the binding patterns of compounds with the highest scores. One hydrogen bond of compound DES was HIS^458^. The existence of intermolecular hydrogen bonds enabled the whole structure of DES to be fixed in hydrophobic cavities on the surface of laccase molecule. Additionally, the interaction between some Pi-Alkyl groups and the existence of carbon-hydrogen bonds also contributed to the stability of the product. The conformation stability between laccase and surrounding free amino acids was mainly maintained by van der Waals, pi-pi T-shaped, Pi-Donor Hydrogen Bond, Alkyl, Pi-Sigma, and Pi-Alkyl interactions. This phenomenon showed a good combination between DES and laccase [59].

## 4 Discussion

The optimization of laccase immobilization conditions is crucial for enhancing operational stability in industrial applications. Immobilized laccase (Fe_3_O_4_@Al^3+^@laccase) displayed maximum activity at pH 5.0 and retained over 65 % of its activity between pH 3–6, compared to free laccase which peaked at pH 4.0. This broader pH tolerance likely arises from the protective effect of the Fe_3_O_4_@Al^3+^ carrier, which stabilizes the enzyme structure. Both free and immobilized laccase showed optimal activity at 40°C, but the immobilized form demonstrated better resistance to temperature fluctuations. Enzyme activity initially increased with substrate concentration, peaking at 1.0 mg/mL, beyond which activity declined due to spatial hindrance. Immobilization time studies showed that 1 h was optimal, as longer times led to structural changes reducing activity. Enzyme kinetics analysis revealed a lower K_m_ value for Fe_3_O_4_@Al^3+^@laccase (0.0073 mM) compared to free laccase (0.0162 mM), indicating a higher substrate affinity likely due to reduced steric hindrance post-immobilization. However, the lower V_max_ of immobilized laccase suggests decreased catalytic efficiency, possibly due to restricted conformational flexibility or active site modifications during immobilization.

Fluorescence quenching experiments indicated a static quenching mechanism between laccase and DES, with a red shift in emission peaks suggesting conformational changes in laccase upon DES binding. Thermodynamic parameters revealed that electrostatic forces and hydrogen bonding dominated the interaction. 3D fluorescence spectroscopy further confirmed DES-induced conformational changes in laccase. TIR analysis confirmed successful laccase immobilization on Fe_3_O_4_@Al^3+^, with characteristic peaks disappearing post-immobilization. SEM and TEM imaging showed spherical Fe_3_O_4_@Al^3+^@laccase nanoparticles (100–200 nm) retaining the carrier's structural and morphological characteristics. Organic solvent studies demonstrated reduced laccase activity in solvents compared to water, attributed to solvent disruption of laccase hydration layers. HPLC analysis showed immobilized laccase degraded DES effectively, with efficiency increasing to 90 % over time before declining, possibly from reduced enzyme activity or limited binding sites. Repeatability experiments found residual activity decreased with cycles, retaining 31.29 % after 10 uses, likely due to enzyme denaturation or shedding during reactions. MEP analysis highlighted DES’s positive character, aiding understanding of laccase-DES interactions. FMO analysis indicated DES’s HOMO-LUMO energy gap, reflecting molecular stability. Molecular docking showed favorable binding of DES to laccase, with interactions like hydrogen bonds and van der Waals forces stabilizing the complex. These findings collectively enhance the understanding of laccase-DES interactions and the practical application of immobilized laccase in bioremediation.

Compared to waste-paper-derived α-Cellulose–Fe₃O₄–CTNs–laccase, which retains 80.7 % activity after 10 cycles but functions optimally only at 30 °C [60], and laccase–TEMPO co-immobilized on Fe₃O₄ nanoparticles, which retains 50 % activity after eight cycles but lacks thermal stability data [61], the Fe₃O₄@Al³ ⁺@laccase system demonstrates a competitive performance profile. Furthermore, Trametes versicolor laccase immobilized on Fe₃O₄-SiO₂-NH₂ retains approximately 60 % of its 2,4-dichlorophenol removal efficiency after seven cycles. However, its optimal operating temperature of 65°C requires higher energy consumption [62]. In comparison, CTAB-KOH-modified biochar-immobilized laccase retains only 45.1 % relative activity after six reaction cycles, while the Fe₃O₄@Al³ ⁺@laccase system retains 56.28 %±1.59 % residual activity after six cycles, and 31.29 %±1.99 % after 10 consecutive cycles, highlighting its superior reusability and operational stability for extended industrial applications [63]. Additionally, while some systems exhibit excellent performance in specific metrics, their fabrication methods are often complex, costly, and associated with limited cycle longevity [40], [64], [65].

## 5 Conclusion

In this study, a novel magnetic nanocomposite (Fe₃O₄@Al³⁺@laccase) was successfully synthesized by immobilizing laccase onto a magnetic support (Fe₃O₄@Al³⁺). The optimal immobilization conditions were determined as 40°C, pH 5, an enzyme concentration of 1.0 mg/mL, and a reaction time of 1.0 h. Under these conditions, Fe₃O₄@Al³ ⁺@laccase achieved 91.71 %±2.95 % degradation of DES within 12 h and retained 31.29 %±1.99 % of its initial enzymatic activity after 10 reuse cycles. Notably, Fe₃O₄@Al³ ⁺@laccase exhibited significantly enhanced stability in organic solvents, which can be attributed to the protective Fe₃O₄@Al³ ⁺ shell. FTIR spectroscopy confirmed successful laccase immobilization, as evidenced by the disappearance of the absorption peak at 3181.98 cm⁻¹ . SEM and TEM analyses revealed no significant changes in surface morphology or crystalline structure after laccase loading, thereby preserving substrate–enzyme interactions. The binding mechanism between Fe₃O₄@Al³ ⁺@laccase and DES was also systematically investigated. Enzyme kinetic parameters (K_m_ and V_max_) of Fe₃O₄@Al³ ⁺@laccase were lower than those of free laccase, indicating higher substrate affinity. This phenomenon was attributed to covalent stabilization at multiple sites or partial rearrangement of the active site of enzyme upon immobilization. Fluorescence spectroscopy revealed that changes in the intrinsic fluorescence intensity of laccase were primarily due to microenvironmental alterations around tryptophan residues. Molecular docking simulations further confirmed favorable interactions between DES and laccase, with a binding free energy of −6.4 kJ·mol⁻¹ . These findings demonstrate that Fe₃O₄@Al³ ⁺ is a promising carrier for laccase immobilization, offering a reusable and efficient platform for degrading phenolic endocrine disruptors such as DES. This approach holds significant potential for environmental remediation and pollutant removal.

## Author statement

All authors approved the manuscript contents and affirmed that this work is not under consideration or published elsewhere. We have no other submission that contains overlapping content with this manuscript. We claim no conflict of interest.

## CRediT authorship contribution statement

**Chenxi Ren:** Data curation. **Tianzhu Guan:** Data curation. **Canfeng Bian:** Formal analysis. **Yining Feng:** Formal analysis. **Qingling Wang:** Visualization, Conceptualization. **Huaxiang Li:** Software.

## Declaration of Competing Interest

The authors declare that there have no conflicts of interest. The manuscript, data, or parts thereof, has not been submitted for possible publication to another journal or that the work has previously been published elsewhere. All relevant data for this article can be found in the manuscript and supplementary materials.

## Data Availability

All data generated or analyzed during this study are included in this manuscript.
