# Supplementary material for: Construction and utilization of Fe3O4@Al3 + immobilized laccase for enhancing organic diethylstilbestrol removal: A multi-spectroscopy and molecular docking investigation
Source: Comput Struct Biotechnol J. 2025 Aug 13;29:236–47. doi: 10.1016/j.csbj.2025.07.044 (PMC12765991; doi:10.1016/j.csbj.2025.07.044)
Supplement: Supplementary file 1 — Supplementary material [file mmc1.docx]

**Research article**

**Construction and utilization of Fe_3_O_4_@Al^3+^ immobilized laccase for enhancing organic diethylstilbestrol removal: a multi-spectroscopy and molecular docking investigation**

**Tianzhu Guan^a,b,c*^,** **Chenxi Ren^b,c^,** **Yining Feng^b,c^,** **Canfeng Bian^b,c^,** **Huaxiang Li^b,c^,** **Qingling Wang^b,c,*^**

^a^ *National R & D Center for edible Fungus Processing technology, Henan University, Kaifeng 475004, China*

^b^ *School of Food Science and Engineering, Yangzhou University, Yangzhou, 225127, China*

^c^ *Key Laboratory of Catering Food Safety and Systematic Monitoring for Jiangsu Province Market Regulation*

* Corresponding authors.

*E-mail addresses:* guantz@yzu.edu.cn (Tianzhu Guan) and wangql891228@163.com (Qingling Wang).

**Supplementary Figure 1.** Standard curve of the fluorescence quenching spectra of laccase and DES system. (A) 305 K, (B) 310K, (C) 315 K.
