# Supplementary material for: Construction and utilization of Fe3O4@Al3 + immobilized laccase for enhancing organic diethylstilbestrol removal: A multi-spectroscopy and molecular docking investigation
Source: Comput Struct Biotechnol J. 2025 Aug 13;29:236–47. doi: 10.1016/j.csbj.2025.07.044 (PMC12765991; doi:10.1016/j.csbj.2025.07.044)
Supplement: Supplementary file 2 — Supplementary material [file mmc2.docx]

**CVs**

- Tianzhu Guan^a,b,c*^

a National R & D Center for edible Fungus Processing technology, Henan University, Kaifeng 475004, China

b School of Food Science and Engineering, Yangzhou University, Yangzhou, 225127, China

c Key Laboratory of Catering Food Safety and Systematic Monitoring for Jiangsu Province Market Regulation

* Corresponding authors.

E-mail addresses: guantz@yzu.edu.cn (Tianzhu Guan)

- Chenxi Ren^b,c^

b School of Food Science and Engineering, Yangzhou University, Yangzhou, 225127, China

c Key Laboratory of Catering Food Safety and Systematic Monitoring for Jiangsu Province Market Regulation

E-mail addresses: rcx1090161416@163.com

- Yining Feng^b,c,^

b School of Food Science and Engineering, Yangzhou University, Yangzhou, 225127, China

c Key Laboratory of Catering Food Safety and Systematic Monitoring for Jiangsu Province Market Regulation

E-mail addresses: 741347156@qq.com

- Canfeng Bian^b,c^,

b School of Food Science and Engineering, Yangzhou University, Yangzhou, 225127, China

c Key Laboratory of Catering Food Safety and Systematic Monitoring for Jiangsu Province Market Regulation

E-mail addresses: biancfyzu@163.com

- Huaxiang Li^b,c,^

b School of Food Science and Engineering, Yangzhou University, Yangzhou, 225127, China

c Key Laboratory of Catering Food Safety and Systematic Monitoring for Jiangsu Province Market Regulation

E-mail addresses: lihuaxiangyu@126.com

- Qingling Wang^b,c,*^

b School of Food Science and Engineering, Yangzhou University, Yangzhou, 225127, China

c Key Laboratory of Catering Food Safety and Systematic Monitoring for Jiangsu Province Market Regulation

* Corresponding authors.

E-mail addresses: wangql891228@163.com (Qingling Wang).
